# The effect of threshold level on bone segmentation of cranial base structures from CT and CBCT images

**DOI:** 10.1038/s41598-020-64383-9

**Published:** 2020-04-30

**Authors:** Luca Friedli, Dimitrios Kloukos, Georgios Kanavakis, Demetrios Halazonetis, Nikolaos Gkantidis

**Affiliations:** 1 Department of Orthodontics and Dentofacial Orthopedics, University of Bern, CH-3010 Bern, Switzerland; 2 Department of Orthodontics and Dentofacial Orthopedics, 251 Hellenic Air Force General Hospital, GR-11525 Athens, Greece; 3 Department of Pediatric Oral Health and Orthodontics, University Center for Dental Medicine - UZB, University of Basel, CH-4058 Basel, Switzerland; 4 Department of Orthodontics, School of Dentistry, National and Kapodistrian University of Athens, GR-11527 Athens, Greece

**Keywords:** Anatomy, Outcomes research

## Abstract

The use of a single grey intensity threshold is one of the most straightforward and widely used methods to segment cranial base surface models from a 3D radiographic volume. In this study we used thirty Cone Beam Computer Tomography (CBCT) scans from three different machines and ten CT scans of growing individuals to test the effect of thresholding on the subsequently produced anterior cranial base surface models. From each scan, six surface models were generated using a range of voxel intensity thresholds. The models were then superimposed on a manually selected reference surface model, using an iterative closest point algorithm. Multivariate tests showed significant effects of the machine type, threshold value, and superimposition on the spatial position and the form of the created models. For both, CT and CBCT machines, the distance between the models, as well as the variation within each threshold category, was consistently increasing with the magnitude of difference between thresholds. The present findings highlight the importance of accurate anterior cranial base segmentation for reliable assessment of craniofacial morphology through surface superimposition or similar methods that utilize this anatomical structure as reference.

## Introduction

Historically, superimpositions of cephalometric radiographs have been used to assess growth and treatment effects on craniofacial morphology^1^. However, dimensional reduction, magnification, distortion, and overlapping of anatomical structures are inherent limitations of 2D imaging^2^. 3D imaging techniques, on the other hand, are free of magnification and overlapping of neighboring structures.

Reported techniques for the superimposition of 3D datasets include landmark-based superimposition, surface-based superimposition, or voxel-based superimposition of form-stable anatomical structures^3–5^. The surface-based technique offers the advantage of easier data handling, processing, storage, and post-processing, and thus, allows for better communication among scientists and easier evaluation of the superimposition outcomes^3^. However, there are potential sources of error including the form of the data, the surface creation and processing (e.g. smoothing, segmentation), the transformation model, and the choice of reference structures^2^.

The anterior cranial base is a standard superimposition reference structure of the craniofacial area^3,6^, due to the anatomical stability of its form since early life stages^7^ and its central location within the craniofacial complex^8^. Therefore, proper imaging of this reference structure is a prerequisite for valid superimposition outcomes.

The image quality of 3D radiographic scans depends on various parameters such as the scanning unit, the examination time, tube voltage and amperage, spatial resolution/voxel size, as well as the field of view (FOV) and the examined object, especially in CBCT scans^9^. CBCT is the radiographic method of choice for dental and maxillofacial surgery patients. It offers less radiation exposure, lower acquisition times, and has a lower cost than conventional CT, in the expense, however, of decreased quality. Furthermore, CBCT imaging produces different voxel values (radiographic densities) for similar structures in different areas of the scanned volume^10–12^.

Thus, the threshold value used for bone segmentation is a critical factor^2^ especially when treating CBCT data^13^ that may present low contrast, inhomogeneity, noise, and artifacts. Ideally, different threshold values should be used for different regions of the head^14^. Currently, however, one threshold value is arbitrarily selected by software and is sometimes manually adjusted by the user. Previous studies have tested the effect of bone segmentation procedures on surface-model accuracy^13–19^, but most did not use actual patient data and none focused on the anterior cranial base.

The primary objective of this study was to explore the effect of varying single threshold values used for bone segmentation of the anterior cranial base, on the produced surface models. Such models are then regularly used for superimposing successive patient head scans to assess craniofacial changes over time. CT versus CBCT data and high versus low quality/radiation data were tested.

## Materials and Methods

### Ethical approval

The study protocol was approved by the Institutional Ethics and Research Committee of the 251 Hellenic Airforce Hospital, Athens, Greece (approval number: 076/10571/16.06.2018). The methods were carried out in accordance to the relevant guidelines and regulations. All participants signed an informed consent prior to the use of their data in the study.

### Sample

**Figure 1:**
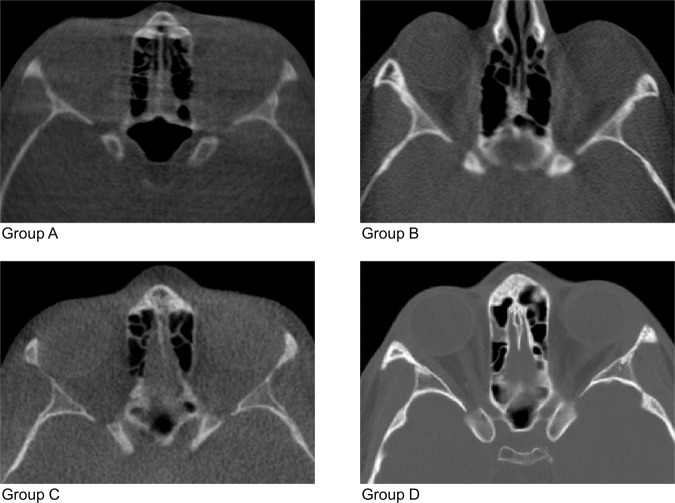
Sample images of each group showing the anterior cranial base region. Images of groups **A**, **B**, and **C** are CBCT scans. Image of group **D** is a CT scan.

The study sample consisted of four groups of pre-existing scans; three groups of 10 large field of view (cranial base to mandible) CBCTs and one group of 10 CTs (including the cranial base). All groups had balanced sex distribution and images were of acceptable quality for craniofacial morphology assessment, as assessed by visual inspection. The images were originally acquired for clinical purposes based on the ALADA (as low as diagnostically acceptable) principle. One CBCT group (A) included lower quality images, whereas two CBCT groups (B and C) included regular quality CBCT images, originally used to assess craniofacial morphology. The CT group (D) comprised high quality images that were generated in a hospital to assess pathological conditions. Detailed information on machines and image acquisition protocols is provided in Table 1 and sample images of each group are shown in Fig. 1.

**Table 1:** Machines and settings used to obtain the images tested in this study.

Machine	Type	Tube voltage (kV)	Tube curent (mA)	Exposure time (s)	Voxel size (mm^3^)	Field of view (mm)
A: ILUMA LFOV (IMTEC Corporation)	CBCT	120	3.8	7.8	0.3	210 × 210 × 140
B: ILUMA LFOV (IMTEC Corporation)	CBCT	120	1	40	0.3	210 × 210 × 140
C: Kavo 3D Exam	CBCT	120	5	3.7	0.4	230 × 230 × 172
D: GE Brightspead 16 asir elite	CT	120	325	9	0.5	250 × 250 × 156

The inclusion criteria used for sample selection were the following:

- 3D radiographic scans of patients 10–14 years of age
- No scans with extensive metal artifacts that might have affected the depiction of the cranial base on the image
- No scans with signs of high noise that would not be originally accepted to assess craniofacial morphology
- Voxel size between 0.25–0.4 mm^3^ for CBCTs and smaller than 0.5 mm^3^ for CTs
- Scans including the entire anterior cranial base.

### Bone segmentation and Surface model generation

The DICOM files of each CBCT and CT scan were imported in Viewbox 4 software (version 4.1.0.1 BETA 64, dHAL software, Kifisia, Greece). One trained operator (L.F.) established a threshold value as “reference” for bone segmentation, as follows: first, the operator selected the range of grayscale values of each radiographic volume, by identifying the minimum (soft-tissue structures) and maximum (hard-tissue structures) value of interest, on the histogram of the scan. Then, any extreme grayscale values, attributed for example to artifacts, were excluded, and the images were reprocessed to increase contrast (Supplementary Figure S1, A and B). The original grayscale range of the voxels of interest, as well as the reference threshold value, of each scan were rescaled on a 0 to 1,000 scale for consistency reasons. To define the reference threshold for each scan, the osseous structures of the anterior cranial base were manually segmented, resulting in two groups of voxels; the anterior cranial base (ACB) voxels and the non-ACB voxels (Supplementary Figure S1, C and D). We disregarded the internal voxels of both groups, i.e. those that were completely surrounded by voxels of the same group, and kept the voxels that had at least one neighbor belonging to the other group. The grayscale values of these voxels, belonging to the interface between the ACB and the surrounding structures, were exported to Microsoft Excel (Microsoft Corporation, Redmond WA, USA). The interface voxels correspond both to bone and soft tissue, and, due to the volume averaging effect^20,21^, they have an intermediate grayscale value. The average grayscale value of the interface voxels was computed and considered the “reference” threshold for anterior cranial base segmentation.

**Figure 2:**
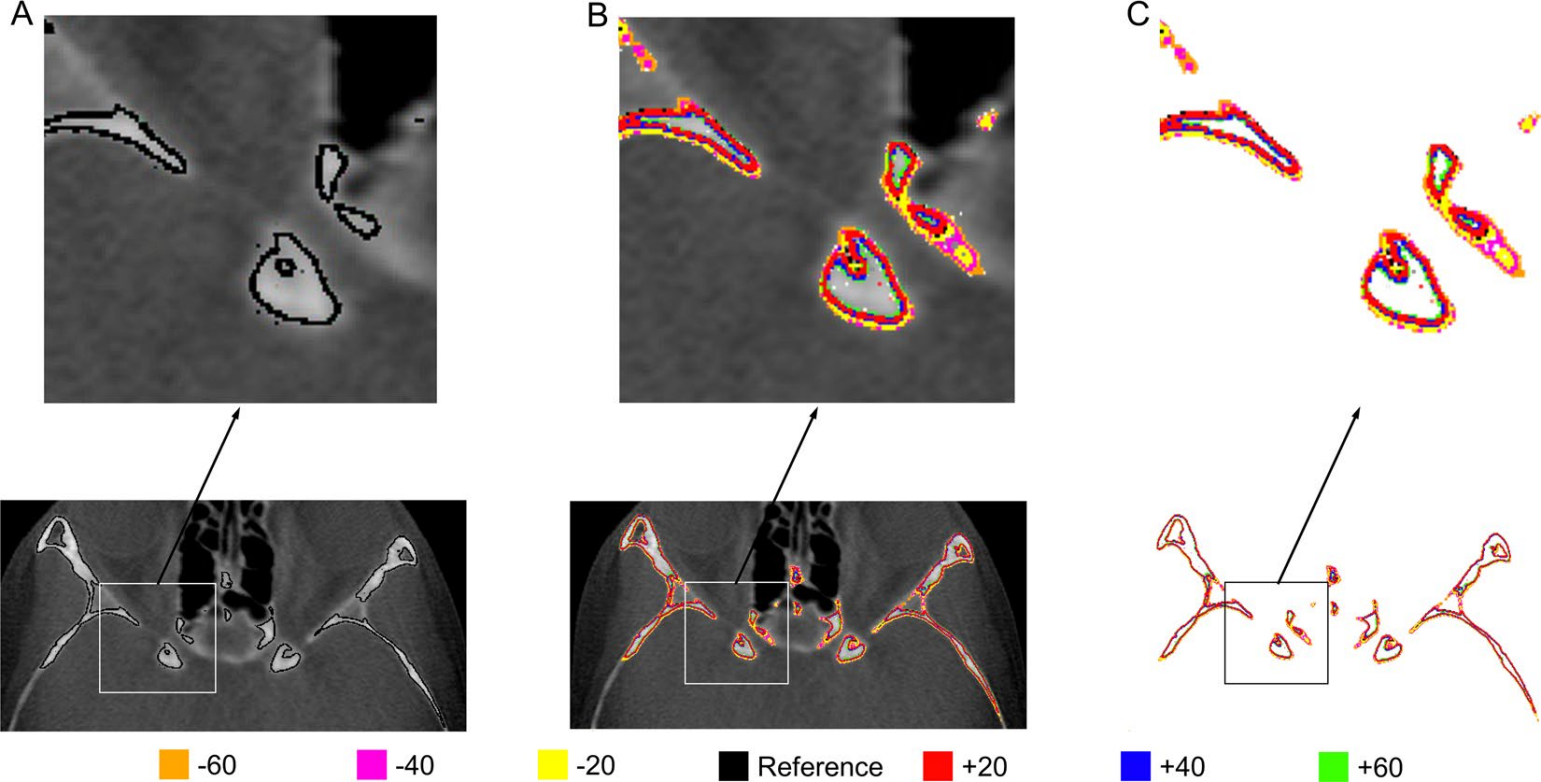
(**A**–**C**) Image of a patient of Group B, showing the selection obtained through each threshold value used to generate the surface models for the study. The colour coding represents the different threshold values. (**A**) Reference threshold value, (**B**) all thresholds, superimposed on the same CBCT slice, (**C**) all thresholds, without background.

**Figure 3:**
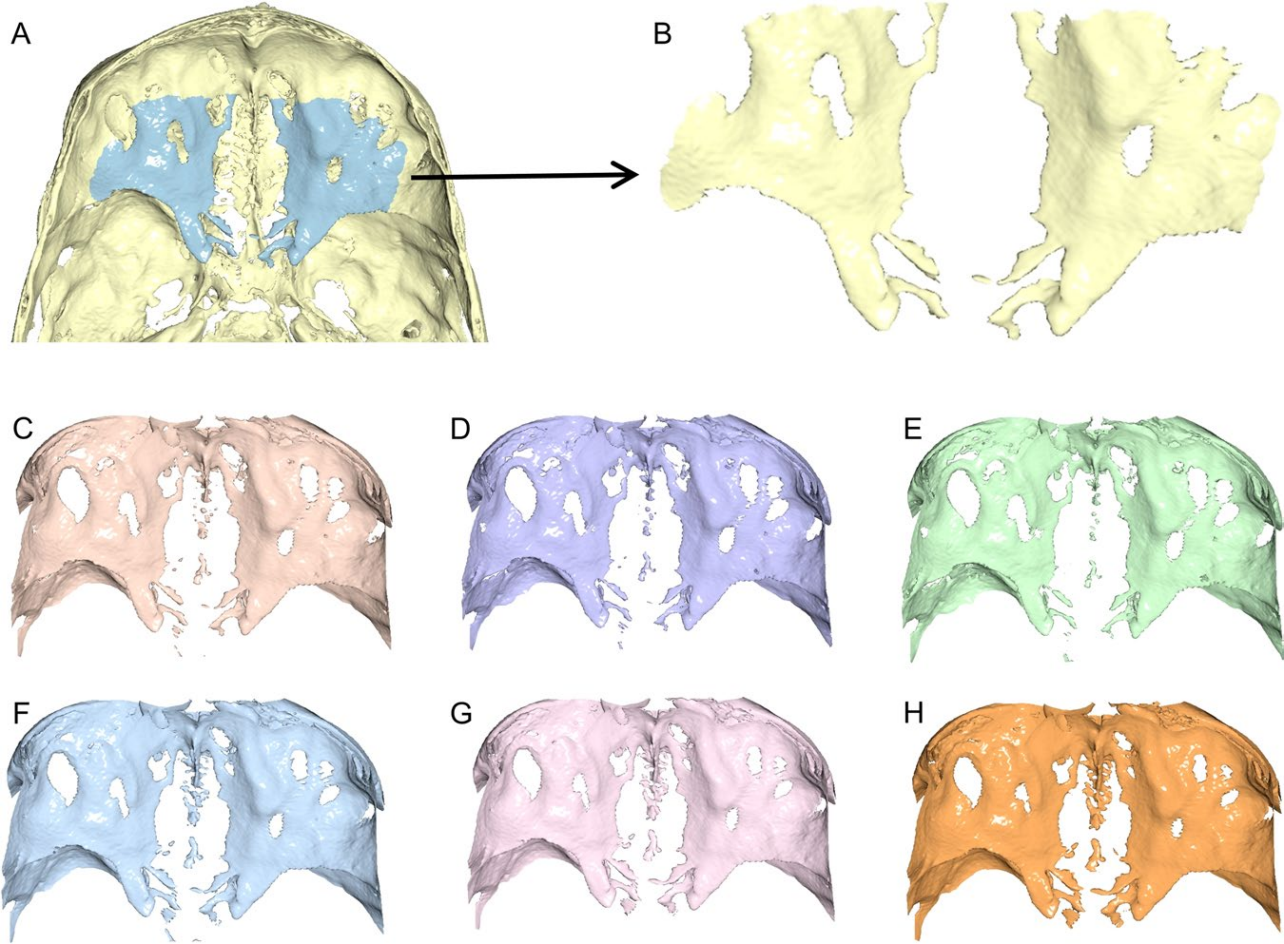
(**A,C**–**H**) Seven anterior cranial base surface models of a CBCT scan from group B extracted using different thresholds (**A**: reference threshold, **C**: +20, **D**: +40, **E**: +60, **F**: −20, **G**: −40, **H**: −60). The blue colour in image A defines the exact anterior cranial base structure that was used in the study (black arrow, **B**). This included the upper surface of the major, central part of the anterior cranial fossa floor, of the sphenoid and the frontal bone, as well as the anterior clinoid processes. The midline structures, especially of the ethmoid bone were excluded.

On the rescaled greyscale range of 0–1,000, the soft tissues in the CBCT images had voxel values in the lower range, up to approximately 400, and bone voxels had values that started from around 260. The observed overlap is expected, mainly due to artefacts of CBCT images^22–24^. We covered most of this range by arbitrarily choosing a total of 6 threshold values, 20 units apart, 3 on either side of the reference threshold, to extract seven anterior cranial base surface models from each scan (reference threshold, +20, +40, +60, −20, −40, −60; Fig. 2). The area of interest tested in the study was selected on the reference surface model that was also considered the reference for superimpositions (Fig. 3). Furthermore, for each scan, the operator visually defined a threshold value, to simulate the usual practice and test its consistency with the manually generated reference value.

### Data generation and superimposition process

The six anterior cranial base surface models were compared to the reference model of each scan in three ways. First, the mean absolute distance (MAD) of each model from the reference was calculated. This variable shows the difference of the models, in their original position in space. The models were dense triangular mesh models, obtained from the CBCT or CT voxel data, using a variant of the marching cubes algorithm^25^ and consisted of approximately 35,000 or 5,500 vertices, respectively. The software measured the distance of each vertex point of one mesh model to the closest point on the second mesh model and calculated the average value, the MAD.

The six models were then superimposed on the reference model, through a variant of the iterative closest point (ICP) algorithm^26^, under the following software settings: 100% estimated overlap of meshes, matching point to plane, exact nearest neighbor search, 100% point sampling, 50 iterations. The rotation and translation required for best fit superimposition of each model to the reference model was measured to describe differences between the original position of the models and the final position obtained after the superimposition. Finally, the MAD between each of the superimposed models from the reference model was measured to test the similarity of the cranial base models, independent of their position in space. Zero translation and rotation and zero MAD prior to superimposition were perceived as no effect of threshold on the subsequent models.

### Statistical analysis

Statistical analysis was carried out with IBM SPSS for Windows (Version 25.0. Armonk, NY: IBM Corp) and the PERMANOVA software^27,28^.

Raw data were tested for normality of distribution through the Kolmogorov-Smirnov and Shapiro-Wilk tests. Evidence of non-normality was present, and thus, non-parametric statistics were applied.

Differences in the measured variables were evaluated using permutational multivariate analysis of covariance (MANCOVA), with factorial mixed or random effects models. Patient was set as a covariate in all cases to account for possible matching and clustering effects. Pair-wise a-posteriori comparisons were performed when significant differences were detected by the multivariate model and further investigation was considered reasonable.

Spatial and morphological differences between the cranial base surface models were assessed before and after the superimposition, respectively. This was performed by testing three factors and their possible interactions: machine (random factor; 4 machines), superimposition status (fixed factor; 2 statuses: before, after), and threshold (random factor: 6 thresholds). Machine and threshold were crossed factors, whereas superimposition was nested in threshold factor. The MAD between the cranial surface models was the testing variable.

Spatial and morphological differences between surface models were also tested using the movement performed by each model, to be superimposed on the reference model, as the testing variable. For this, two crossed factors and their possible interactions were analyzed: machine (random factor; 4 machines) and threshold (random factor: 6 thresholds). All vectors of positional change of each surface model were considered as dependent variables (6 vectors: x-lateral movement, y- anteroposterior movement, z-vertical movement, x-rotation, y-rotation, z-rotation).

Permutational MANCOVA was done on Euclidean distances calculated from raw data. The P-value was calculated on raw data through permutation of residuals under a reduced model, with 999 random permutations. In cases when there were few unique permutations possible Monte Carlo asymptotic p-value was used instead^27^ (PERMANOVA).

In all cases, a two-sided significance test was carried out at an alpha level of 0.05. Bonferroni correction was applied for pairwise a-posteriori multiple comparison tests that were performed in a paired manner through Friedman’s or Wilcoxon’s signed-rank tests.

### Method error

The whole surface model generation and superimposition process was repeated by the same operator (L.F.) after a 4 week-period, for 12 randomly chosen radiographic scans (3 from each group).

## Results

### Method error

The median difference between repetitions in the range of grayscale values selected to be rescaled from the original histogram was 1 (range: −28, 43; Wilcoxon’s signed rank test, p = 0.289), which was considered negligible compared to the original extent of relevant grayscale values (median: 2175; range: 1842, 2829).

The difference between repeated visually defined thresholds was also very small (median: −4; range: −23, 18) and not significant (Wilcoxon’s signed rank test, p = 0.530).

Differences between repeated manually defined reference thresholds were also small and not significant before (median: 1; range: −64, 72; Wilcoxon’s signed rank test, p = 0.784) and after rescaling (median: −4.8; range: −23.5, 42; Wilcoxon’s signed rank test, p = 0.583).

The differences between repeated calculations of the MAD values between models prior to and following superimposition on the reference model, were consistently identical. The same was true for the rotation and translation required for best fit superimposition of each model to the reference model.

### Visually vs. manually defined reference threshold

The median difference between the visually and manually defined thresholds can be considered small (median: 36.5, range: −61, 253), when compared to the original extent of the grayscale values (median: 2175; range: 1842, 2829), but it was statistically significant (Wilcoxon’s signed rank test, p = 0.001). The median absolute difference was of similar extent (median: 44.5, range: 1, 253). Supplementary Figure S2 shows colour maps of the surface models that represent the minimum, median and maximum differences.

### MAD of the original models obtained through different thresholds from the reference model

Multivariate tests showed a significant effect of all three factors (machine, threshold, superimposition) on the results, as well as significant interactions of the machine, with the threshold and the superimposition factor (p < 0.05, Table 2).

**Table 2:** Non parametric MANCOVA on MAD of the models obtained through different thresholds from the reference model, by different machines, thresholds, and superimposition statuses. Three factors and their interactions were analyzed having “patient” as a covariate: machine (random factor; 4 machines), threshold (random factor; 6 thresholds) and superimposition status (fixed factor; before and after superimposition). Machine and threshold were crossed factors, whereas superimposition was nested in threshold factor. *p < 0.05.

Distance factor	df	F	P(perm)
Covariate	1	206.3	0.001*
Machine	3	8.6	0.002*
Threshold	5	40.8	0.001*
Superimposition (Threshold)	6	3.4	0.024*
Machine × Threshold	15	3.3	0.001*
Machine × Superimposition (Threshold)	18	6.1	0.001*
Residual	431		
Total	479		
Comparison^a^	P		
A vs. B	0.005*		
A vs. C	0.005*		
A vs. D	0.091		
B vs. C	0.060		
B vs. D	0.457		
C vs. D	0.645		

^a^Tests among levels of the factor Machine. A, B, C, and D correspond to the four radiographic machines tested in the study.

**Figure 4:**
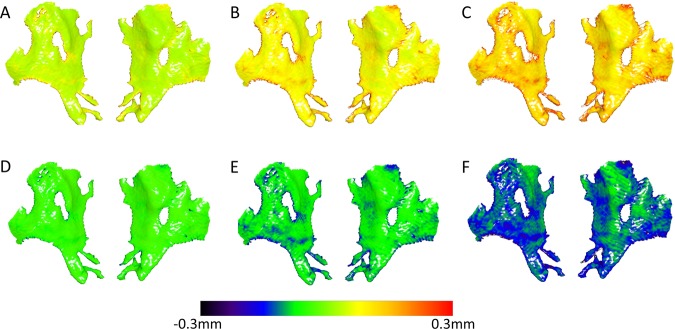
Colour maps showing the differences (distances) of the anterior cranial base models produced through various thresholds (**A**:+20, **B**: +40, **C**: +60, **D**: −20, **E**: −40, **F**: −60), from the reference model, for a single CBCT image of Group B, before superimposition.

**Figure 5:**
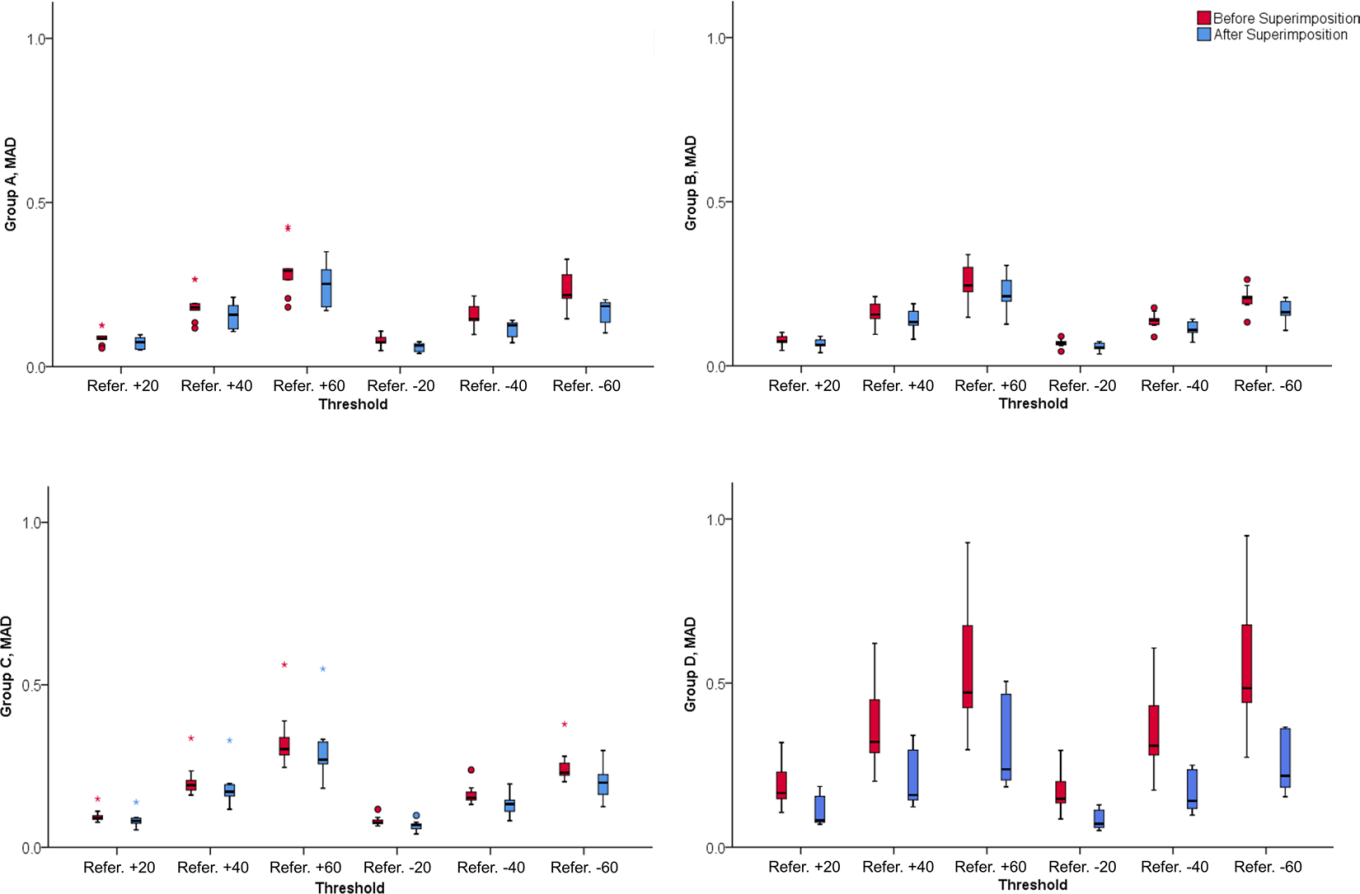
Box plots showing the MAD (mm) of all surface models of each group obtained with the different thresholds from the reference model, before and after the best fit superimposition of each model with the reference (Refer.).

There were detectable MADs between the original (prior to superimposition) models segmented from the DICOM files through different threshold values and the reference model, for all the machines tested. The MAD values were consistently below 0.5 mm for all CBCT machines (median: 0.15, range: 0.04, 0.56 mm; within threshold category Wilcoxon signed rank tests between CBCT groups, P > 0.005). In general, threshold effect was higher for the CT machine, reaching values higher than 0.5 mm in certain cases (median: 0.31, range: 0.09, 0.95 mm; within threshold category Friedman tests, P < 0.005, Wilcoxon signed rank tests between the CT with the CBCT groups, P < 0.005). The distance between the models, as well as the variation within each threshold category, consistently increased with an increase or decrease of the threshold value used in each case (within machine group Friedman test: P < 0.005, Wilcoxon signed rank test: P < 0.005; Figs. 4 and 5).

**Figure 6:**
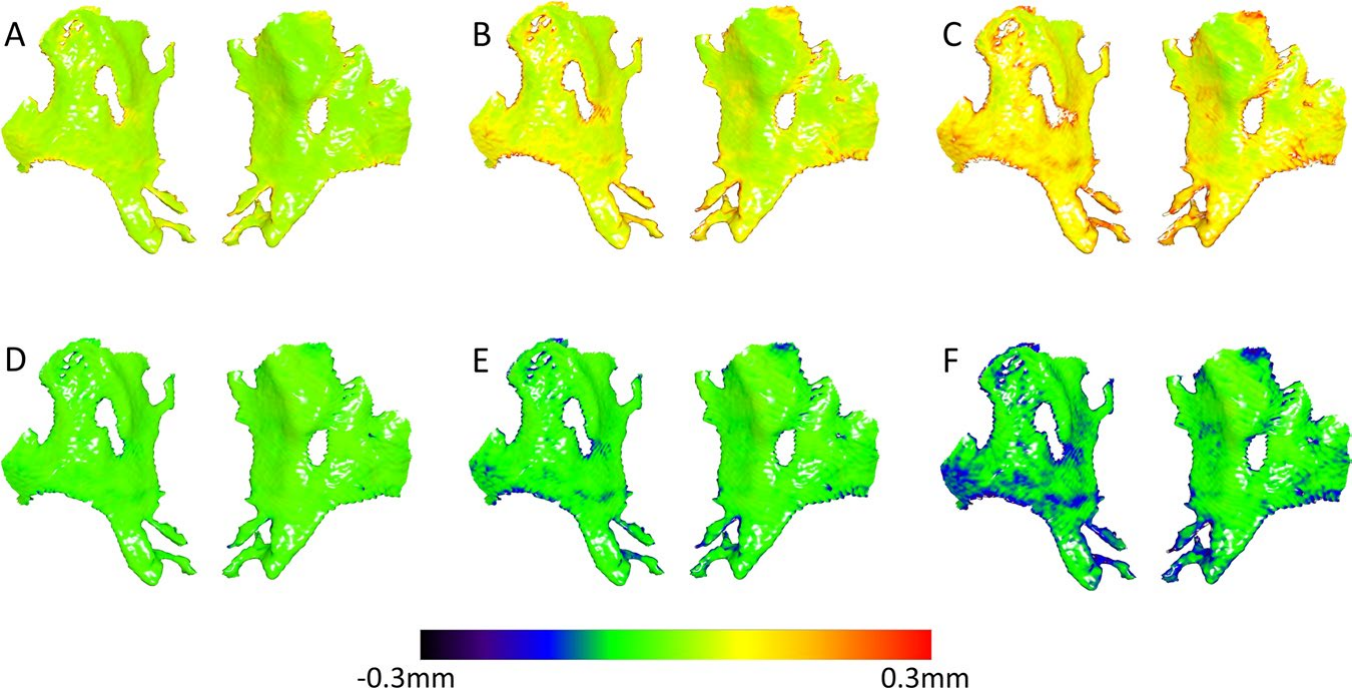
Colour maps showing the differences (distances) of the anterior cranial base models produced through various thresholds (**A**: +20, **B**: +40, **C**: +60, **D**: -20, **E**: −40, **F**: −60), from the reference model, for a single CBCT image of Group B, after best fit superimposition.

Following the best-fit superimposition of the reference surface model to the models obtained through different thresholds, there were still detectable differences in the obtained MAD values for all the machines tested in the study. For the three CBCT machines, the MAD values were slightly decreased compared to those calculated before the best-fit registration (median: 0.13, range: 0.04, 0.55 mm; within threshold category Wilcoxon signed rank tests between CBCT groups, P > 0.005). On the contrary, the CT machine presented considerably reduced MAD values (median: 0.16, range: 0.05, 0.51 mm), reaching similar levels to those of the CBCT groups (within threshold category Friedman tests, P > 0.05). Both, CT and CBCT machines had analogous effects to the magnitude of difference between thresholds and the reference threshold, in a way similar to that prior to superimposition. The higher the difference between thresholds the higher the MAD reduction following the superimposition (within machine group Friedman test: P < 0.001, Wilcoxon signed rank test: P < 0.005; Figs. 5 and 6).

### Positional change of the original models obtained through different thresholds, after their superimposition with the reference model

**Figure 7:**
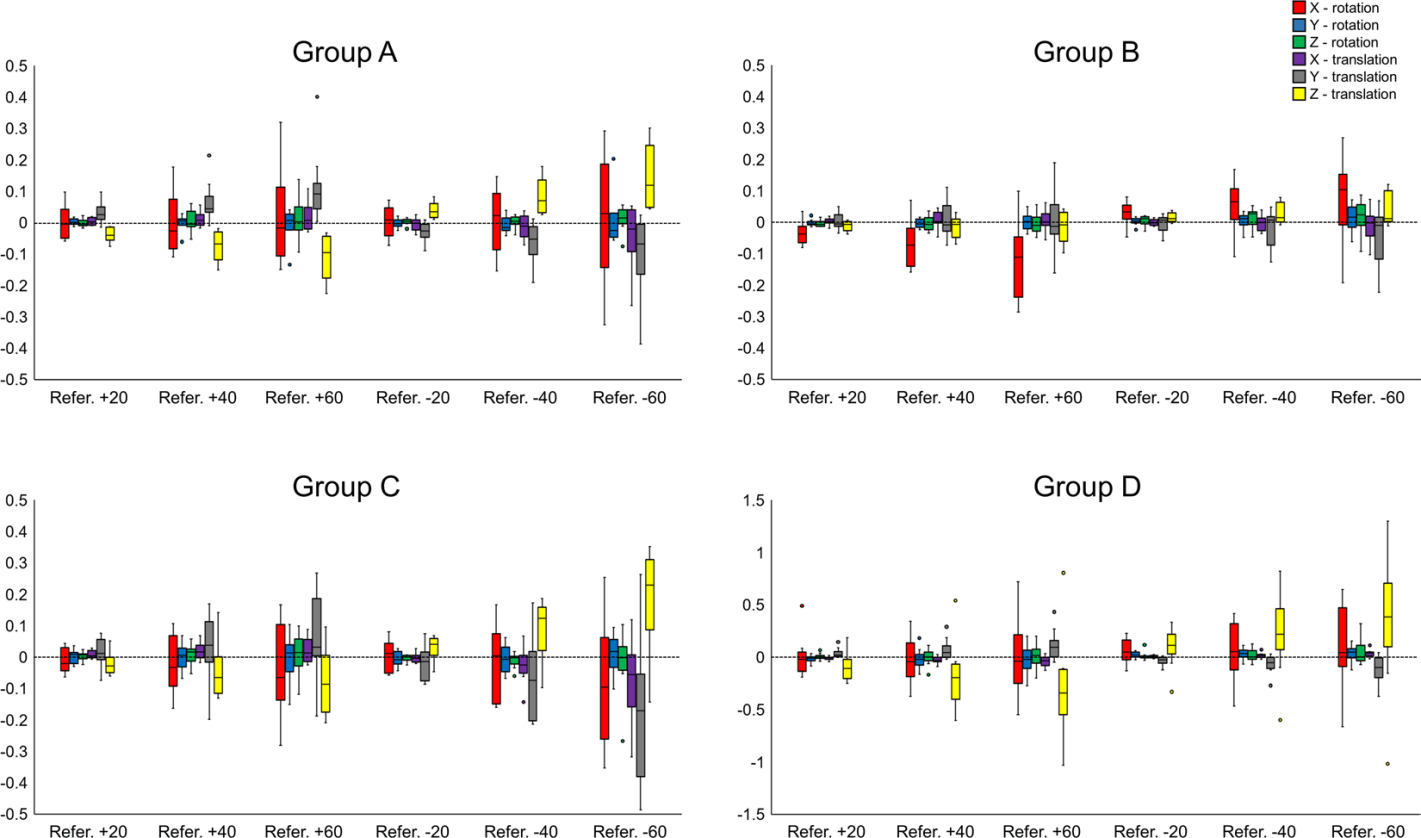
Box plots showing the positional change of the original models (rotation: °, translation: mm) obtained through different thresholds that occurred after their superimposition with the corresponding reference model (X - axis: lateral, positive right; Y - axis: anteroposterior, positive posterior; Z - axis: vertical, positive up; Refer. Reference).

Multivariate tests showed that threshold level was the only factor that exerted a significant effect on the movement required to register each anterior cranial base model to the reference model (p < 0.05, Table 3). Group D showed larger movements, which was in accordance to the outcomes reported above. For all groups, all movements consistently tended to increase as the difference of the individual threshold values from the reference threshold increased. For all four machines, there was a slight tendency for models produced using thresholds smaller than the reference, to show slightly larger change from their original position to that obtained after superimposition with the reference model. Vertical linear movements and anteroposterior rotational movements consistently reached the largest values for all machines (Fig. 7).

**Table 3:** Non parametric MANCOVA on positional changes (movement of the original models obtained through different thresholds, required to superimpose them with the reference model) by different machines and thresholds. Two crossed factors and their interactions were analyzed having “patient” as a covariate: machine (random factor; 4 machines) and threshold (random factor; 6 thresholds). *p < 0.05.

Movement factor	df	F	P(perm)
Covariate	1	1.059	0.337
Machine	3	0.836	0.317
Threshold	5	5.226	0.001*
Machine × Threshold	15	1.189	0.184
Residual	215		
Total	239

## Discussion

To our knowledge, this is the first study to test the outcome of single-threshold based bone segmentation on the anterior cranial base surface model. The anterior cranial base is a standard superimposition reference area used for the assessment of craniofacial morphology, in various disciplines. Here we showed significant effects of the used threshold that were also related to the type of images (CT or CBCT). This might have important implications for the surface superimposition outcome of serial 3D radiographic images that are registered on the cranial base, since small inconsistencies in the superimposition reference areas have been shown to considerably affect the superimposition outcomes^29^.

The results showed that the origin of the data is a significant contributing factor. CT provides high quality images but the high radiation dose, cost^30^, and limited accessibility restricts its use in routine dentistry. CBCT scans offer adequate quality images for diagnosis, treatment planning and progress/outcome evaluation at lower cost and radiation dose. However, an important limitation of CBCTs stems from the way the volume is generated and reconstructed, which differs from that of a CT machine. In CBCTs, the greyscale value of a specific voxel is affected by its position within the image volume, meaning that two voxels with identical tissue density, located at different positions in the scan, might not have the same grayscale value in the reconstructed image. Interestingly, this was favorable for the effect that thresholding had on the segmented models, because the variation in thresholds in CBCTs, affected less the subsequent models, as compared to CT images. However, it should be noted here that bone segmentation through a single threshold is more straightforward in CT images, due to the correspondence of grayscale values to Hounsfield units. Therefore, the selection of a single threshold value on an actual CT scan is more accurate and this was also evident from the repeated selections performed in the present study.

Other factors that may affect image quality are related to the radiographic machine, the scanning parameters, the reconstruction algorithm, FOV, image contrast, signal to noise ratio and patient-specific issues^12,22,31,32^. To be able to generalize our findings, we tested three CBCT machines and images that were obtained under different settings and were of different quality. Indeed, the results were robust for all CBCT images, suggesting that data obtained with lower radiation protocols might not be less reliable in terms of superimposition outcomes. Data from one CT machine were also assessed to include an additional comparison group of a different type and this had a significant effect on the results.

Prior to superimposing the various models on the reference one, the CT derived models showed larger MAD values compared to the CBCT derived models. This could be attributed to the more uniform effect of thresholding on the CT images, leading to higher differences in the spatial position of the segmented models. On the other hand, on the CBCT images, this effect was limited. The non-correspondence of Hounsfield units to greyscale values obtained from a CBCT scan, as well as the limitations of CBCT images that were discussed previously might be related to this finding, which seems, however, to be favorable for the outcomes in this respect. Another possible explanation could be the slightly larger voxel size of the CT compared to the CBCT images. Following the superimposition of the models with the reference surface model, there were still significant deviations from zero in the MAD values obtained from all the machines tested, but the reduction of MAD values was higher for CT images, reaching values similar to those obtained from the CBCT images. The fact that anteroposterior rotations consistently reached the highest values could be due to the porosity of anterior skeletal structures, where a higher threshold effect can be expected in comparison to posterior sites that present denser bone^5,33^.

The findings of the present study are important not only when considering surface-based registration on the anterior cranial base, but also for outcome assessment following landmark- or voxel-based superimposition. When evaluating pairs of models to assess changes that occurred between two time points, the effect of thresholding is present twice; once on the first and once on the second model^5^. Thus, it is a factor that may artificially distort the morphology of the cranial base structures, affecting the outcome of a surface-based superimposition. It might also act in addition to other factors that could affect the surface model morphology over time, such as those related to image inaccuracies or to changes due to growth or aging.

Our sample consisted of individuals aged 10–14 years of age, which are still growing, but not at the anterior cranial base structures, where growth ceases early in development^7^. This sample was selected because it represents the age group of the typical orthodontic patient; however, since the anterior cranial base structures are already fully formed at this age, similar results would be expected in an adult sample.

Surface models do not contain any volumetric data that are originally available after a 3D radiographic examination, but use 3D surface data instead. While this leads to a reduction of information, on the other hand it allows for processing only the most clinically relevant data. Therefore, handling and processing, as well as data storage, become faster and easier. The 3D model of a patient can be superimposed on other such models of the same or other patients to provide reliable outcomes^3^. The surface-based registration technique that was used in this study is well tested^3,4,34^ and has been previously shown to work properly under similar designs^5,35,36^. This was confirmed here by the perfectly reproducible results through repeated superimpositions. The landmark-based registration largely depends on the accuracy of landmark identification. When few landmarks are used, it is faster and easier, but then small mistakes can have a big effect^3,37^. The voxel-based technique could be an alternative that does not need bone segmentation^5^. However, for the visualization and the detailed assessment of superimposition outcomes segmentation and surface model creation is still required^5^.

Manual bone segmentation of every single CBCT image/section could be a solution to the single-threshold based segmentation techniques, but it is considered unrealistic since it would require several hours to be performed in a single dataset and would still be prone to error^14^. Alternatives to overcome this problem have been suggested in the literature^14,38,39^, but the single threshold approach is still the standard process offered by most relevant dental software for simplicity reasons. The range of grayscale values tested in the present study was verified through visual inspection of several datasets and, based on the generated surface models, was considered to be realistic in terms of potential selection by an operator or a software. The differences between manual and visually defined reference thresholds, as well as between repeated threshold selections, were within the range and comparable to the different thresholds tested in the present study, confirming the applicability of our results in actual conditions. Similar intra- and inter-operator differences in the selected thresholds were also exhibited in a recent relevant study^5^.

One limitation of the present study is the fact that no gold standard (true model) was used. One threshold value was manually selected as the reference value for each scan. Although this served the aim of the study, the used reference model might still be prone to errors, as the voxels to extract the cranial base surface model were chosen in a subjective manner. A gold standard model could be a high quality 3D surface scan of the anterior cranial base of a dry skull. However, this would not fully represent the clinical reality of a regular CBCT or CT scan of a patient’s head, where the true model is impossible to be obtained. Another limitation of the study is that three CBCT machines were tested and the possibility of obtaining different results on images from other machines, cannot be excluded. Furthermore, only one operator, trained from an experienced practitioner, ran all the tests. Inter-examiner reliability was therefore not tested, but intra-examiner reliability showed satisfactory results. Finally, this study was performed on 3D images of growing patients. We expect similar results in adult patients, but further studies might be needed to confirm this.

## Conclusion

The level of the single threshold used to perform bone segmentation of cranial base structures from 3D radiographs has a significant impact on the subsequent surface models; with changes being affected by image type. Threshold effects are related to spatial positioning of the surface model and to its form. Effects were more uniform for the CT scans, mainly affecting the position of the surface models in space. The non-uniform effects observed on the CBCT data reflect limitations of this imaging modality related to grayscale values and tissue density. They were similar for different CBCT images and lead to restricted effects of thresholding on the spatial position of the subsequent models. Both for CT and CBCT machines the effects were in accordance to the magnitude of difference between thresholds.

The findings of the study might have important implications for the validity of 3D superimposition outcomes when surface models extracted through a single threshold are used to superimpose serial patient images or to demonstrate changes that occurred during time.

## Supplementary information

Supplementary Figures.

## Data Availability

All data are available in the main text or the extended data. The protocols and datasets generated and/or analyzed during the current study are available from the corresponding author on reasonable request.
