# Supplementary material for: The effect of threshold level on bone segmentation of cranial base structures from CT and CBCT images
Source: Sci Rep. 2020 Apr 30;10:7361. doi: 10.1038/s41598-020-64383-9 (PMC7193643; doi:10.1038/s41598-020-64383-9)
Supplement: Supplementary file 1 — Supplementary Figures. [file 41598_2020_64383_MOESM1_ESM.docx]

**Supplementary material**

**The effect of threshold level on bone segmentation of cranial base structures from CT and CBCT images.**

Luca Friedli, Dimitrios Kloukos, Georgios Kanavakis, Demetrios Halazonetis & Nikolaos Gkantidis


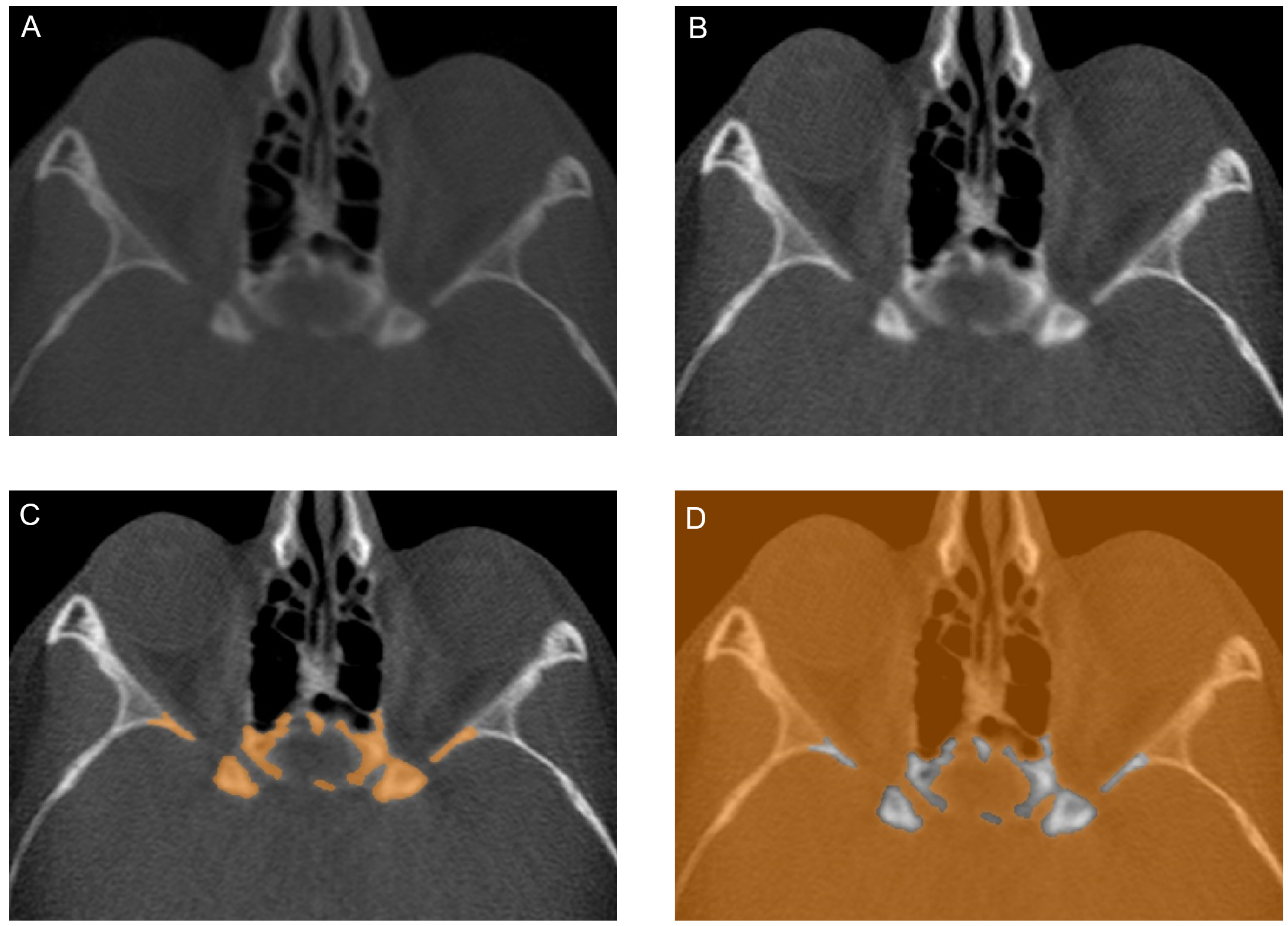


**Supplementary Figure S1.** Images of a CBCT scan from group B, before (A) and after processing (B), through the exclusion of extreme grayscale values, attributed to artifacts. These were extreme intensities originating from metal appliances, such as orthodontic appliances or tooth crown and bridge restorations, or amalgam fillings. The excluded structures were visualized through single thresholding on the original histogram and were confirmed prior to removal. Following, the voxels illustrating the anterior cranial base structures of interest were manually selected (orange colour, C) and the grayscale values of those voxels located at the outer margins of this selection were exported. The selection was inverted (D) and the latter was repeated. The average of the exported values defined the reference threshold. In total 30 to 40 slices were used for each CBCT scan and 15 slices for each CT scan.


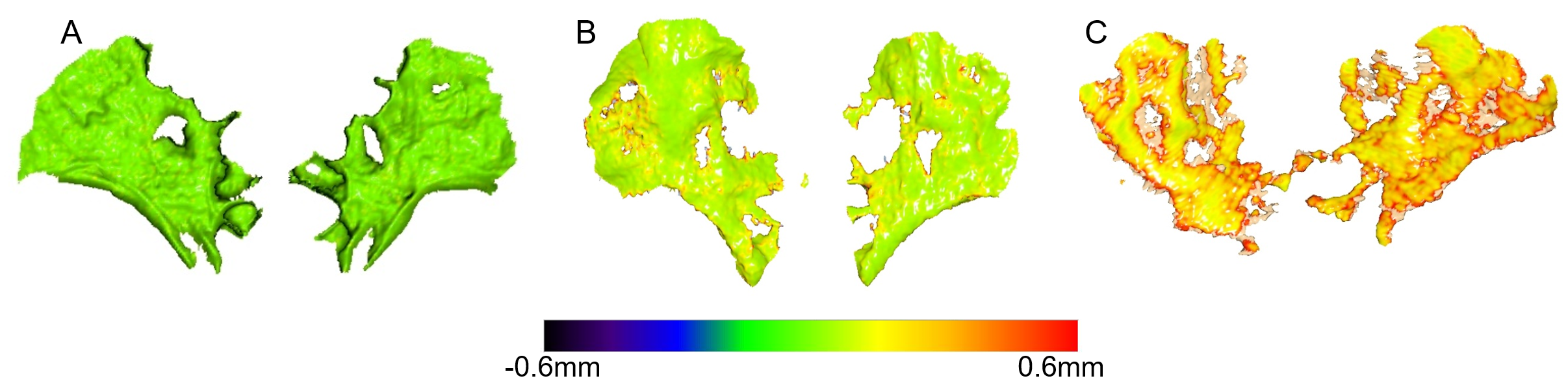


**Supplementary Figure S2.** Colour maps showing the difference (distance) of the surface models obtained using the manually vs. the visually defined reference threshold. A. minimum difference (MAD) observed in a CT image of Group D, B. median difference (MAD) observed in a CBCT of Group C, C. maximum difference (MAD) observed in a CBCT of Group A.
